# Immunohistochemical profiling of the tumour microenvironment in borderline and malignant ovarian tumours in young women

**DOI:** 10.3892/ol.2023.13763

**Published:** 2023-03-15

**Authors:** Danielle O'Neill, Kirstie Rice, Anjali Bhatnagar, Daniel Kearns, Fedor Berditchevski, Alaa El-Ghobashy, Abeer M. Shaaban

**Affiliations:** 1 Department of Gynaecological Oncology, The Royal Wolverhampton NHS Trust, New Cross Hospital, Wolverhampton, West Midlands WV10 0QP, UK; 2 Department of Cellular Pathology, The Royal Wolverhampton NHS Trust, New Cross Hospital, Wolverhampton, West Midlands WV10 0QP, UK; 3 Department of Cellular Pathology, University Hospitals Birmingham NHS Foundation Trust, Queen Elizabeth Hospital Birmingham, Edgbaston, Birmingham B15 2GW, UK; 4 Institute of Cancer and Genomic Sciences, University of Birmingham, Edgbaston, Birmingham B15 2TT, UK

**Keywords:** TILs, borderline ovarian cancer, ovarian cancer

## Abstract

Ovarian cancer is a major cause of cancer-related deaths in women. Our previous study highlighted the interaction between cancer cells and the host immune response in solid cancers. The present study aimed to analyse the proportion, density and distribution of T and B lymphocytes within the tumour and surrounding stroma, and their prognostic significance in young women with borderline and malignant ovarian surface epithelial tumours. Full clinicopathological and outcome data were collected for 57 women aged <50 years diagnosed between January 2010 and December 2015. Representative tumour sections were stained for CD3 (T cells) and CD20 (B cells) and tumour-infiltrating lymphocytes (TILs) were scored following the TILs Working Group Recommendations and described as stromal, intra-tumoural, lymphoid aggregates and touching lymphocytes. Data were statistically analysed and the association with clinicopathological variables was assessed. The median age was 41 years and the most common histological type was serous carcinoma (n=21). The risk of malignancy index was a significant predictor of ovarian cancer diagnosis (P<0.05). A total of 15 out of 34 patients with cancer died. There was significantly greater stromal infiltration of CD3 and CD20 TILs (P=0.01 and P=0.03, respectively) and higher intratumoral CD20 expression in ovarian epithelial cancers compared with borderline tumours. The highest CD3 stroma count and density were observed in serous carcinoma, which also exhibited the highest numbers of CD3 and CD20 aggregates. There was no statistically significant difference between touching lymphocytes and tumour histological subtype. There was no significant association between TIL expression and patient survival. The count, distribution and density of T and B lymphocytes in ovarian tumours varied depending on tumour type and invasiveness. Their topographic distribution within the tumour and surrounding stroma did not impact prognosis in young women with ovarian cancer. TIL analysis in an older age group of women with ovarian tumours is ongoing to determine its potential prognostic significance.

## Introduction

Ovarian cancer is the eighth most common cancer affecting women world-wide (1) and has a UK incidence of over 7,400 cases per year (2). Approximately 70% of women present at an advanced stage with evidence of metastatic disease (3,4). More than 80% of women with stage 3 and 4 disease respond to surgical debulking and chemotherapy (5), however recurrences tend to occur within 22 months. The overall 5 year survival rate is poor (27%) (4,6).

It was not until 2003 that Zhang *et al* (7) demonstrated the association between tumour-infiltrating T cells (CD3+) and prognosis in ovarian cancer. They showed a 5-year survival of 38% in cancers with CD3+ infiltration compared to 4.3% in those where such infiltrates were not evident. It also significantly affected progression-free survival and correlated with delayed recurrence and delayed death. Tumours with high T cell infiltration were more likely to achieve complete cytoreduction (7).

The relevance of TILs has been highlighted in several other solid cancers such as breast and liver (8,9). Additionally, it is not simply the presence or absence of TILs that is of importance, it is their location within the tissue that is becoming of interest. A study by Sato *et al* (10) highlighted the importance of identifying the precise in situ localisation of the TILs suggesting that their topographic location within an ovarian tumour could be of significance to the outcome. This has also been explored by Toss *et al* (11) in breast carcinoma. It is known from prior studies that the presence of TILs in invasive breast cancer positively augmented the effect of chemotherapy mainly in triple-negative cancers (12), prompting the publishing of guidelines recommending TILs evaluation.

Many studies have demonstrated that a lack of TIL infiltration is associated with a poorer outcome in many solid cancers, however the population used in these amongst ovarian cancer studies tends to be older women with a median age in the fifth or sixth decade of life. A meta-analysis performed on TILs in ovarian cancer identified ten suitable studies and the mean age of patients was between 55 and 62 (13).

The significance of TILs in young women with ovarian cancer is yet to be explored. Therefore, this study was conducted to analyse, in detail, the expression and topographical distribution of CD20 and CD3 tumour-infiltrating lymphocytes and their impact on prognosis or response to therapy in ovarian cancer women under the age of 50. Comparisons were drawn between histologically borderline tumours and frankly invasive carcinomas.

## Materials and methods

### Study cohort

This was a retrospective study of women under the age of 50 who underwent surgery for a borderline or invasive ovarian tumour between 1st January 2010 and 31st December 2015 at the Royal Wolverhampton NHS Trust, Wolverhampton, United Kingdom. Ethical approval was sought and granted by the West Midlands-Black Country NRES Committee (07/Q2702/24). Women aged less than 50 were specifically chosen because this age group is under-represented in the current literature and may demonstrate a different pathophysiological course in comparison to women of an older age.

The patients were identified by searching the clinical and pathology databases. Clinicopathological data including patients' age, tumour type, disease stage, management, recurrence and survival were retrieved from case notes and electronic records. Data including overall survival and disease specific survival was collected till 1st December 2019. Information was gathered on standard treatment regimens but data on complementary and alternative medicine was not available and has not been included. Women received standard therapy as per local guidance so would have received the same treatment in accordance with their diagnosis. The influence of any additional treatment factors within this cohort would be marginal and so are likely representative of the general patient population.

### Immunohistochemistry

**Figure 1.**
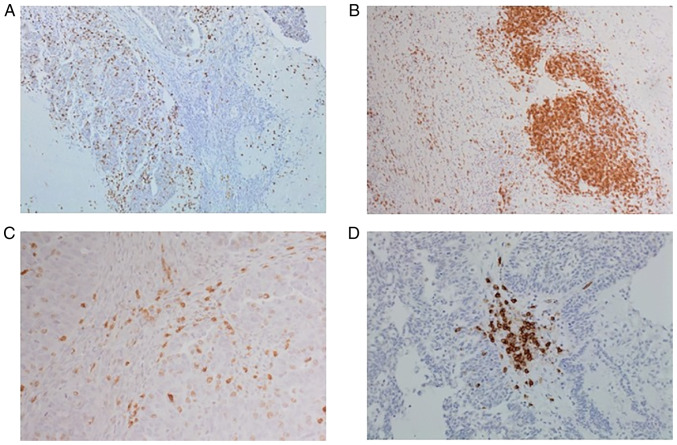
Representative sections of CD3 and CD20 immunohistochemistry in ovarian cancer. Images were captured using an Olympus BX 51 microscope (Olympus Corporation). (A) Stromal tumour-infiltrating CD3 lymphocytes in ovarian mucinous carcinoma (magnification, ×100). (B) CD20-positive stromal tumour-infiltrating lymphocytes in ovarian serous adenocarcinoma (magnification, ×200). (C) CD3-positive touching lymphocytes (magnification, ×400). (D) CD3-positive lymphoid aggregate in ovarian serous adenocarcinoma (magnification, ×100).

Slide review and histological selection of representative full face formalin fixed paraffin wax embedded tumour blocks of the primary treatment naïve tumours was performed. Representative 4 microsections were prepared and immunohistochemically stained for CD3 and CD20 using the Dako Link autostainer using ready-to-use CD3 and CD20 antibodies following the manufacturer's instructions. CD3 staining was completed utilising the Agilent anti-human CD3 polyclonal ready-to-use antibody (product GA503, CE-IVD). CD20 staining was completed utilising the Agilent anti-human CD20 ready to use antibody, clone L26 (product GA604, CE-IVD). Both assays were completed on the Agilent OMNIS platform and visualised with the EnVision FLEX DAB detection kit. The CD3 assay is a pan T-cell marker well suited to labelling reactive T-cells in tissue with lymphoid infiltrates, the CD20 assay reacts with an intracytoplasmic epitope localised on the CD20 antigen and labels cells of the B-cell lineage. Both assays reliably label human B-cells or T-cells in histological tissue preparations. Antibodies were used as per their published protocol. Sections were anonymised using unique identification numbers by a third party and assessors were blinded to the patient and clinical data. Slides were scored manually by one investigator (DO) overseen by a pathologist (AMS). Guidelines produced by the International Immuno-Oncology Biomarker Working Group (8) aim to standardise the assessment of TILs in solid tumours and this guidance was applied to counting the TIL populations in our samples. Briefly, the number of positively stained CD3 or CD20 cells present in ten high-power fields (HPFs) was counted and average per HPF calculated. The percentage infiltration of TILs representing the area occupied by the immune cells in relation to the total stromal area present was also estimated for each patient following the guidelines of the International ImmunoOncology Group (8). Representative slides of CD3 and CD20 IHC staining are shown in Fig. 1.

### Scoring of TILs expression

Intratumoural lymphocytes were defined as those in direct contact with tumour cells or within the tumour itself. The stromal lymphocytes were defined as those lying within the tumoural stroma. Hotspots representing regions with highest densities were recorded if present. Touching lymphocytes were defined as the number of lymphocytes touching the basement membrane or within one lymphocyte cell diameter from the basement membrane as previously described and are shown in Fig. 1C (11). This method was shown to have minimal intra-observer variance and good concordance (11). Lymphoid aggregates (well defined, densely populated areas of lymphocytes within the stroma) where present, were also scored and are shown in Fig. 1D. Any discrepancies were subsequently reviewed by both assessors and a consensus was reached.

### Statistical analysis

Statistical analysis was performed using SPSS statistical software version 26 (IBM Corp.). Normally distributed data are presented as the mean ± standard deviation. For data without normal distribution, such as age, median values (with interquartile range) are presented. Chi-squared analysis (χ^2^) was used to correlate patient outcome (dead vs. alive) with tumour type. The nonparametric independent samples median test [Mann-Whitney-U Wilcoxon (rank sum) test (MWW)] and Kruskal-Wallis test (KWt) were performed to compare the means and medians of the variables, including age, CD3, CD20, touching lymphocytes count, aggregates and hot spot counts among different groups. Spearman's rank correlation coefficient (r_s_) was used to correlate continuous variables of biomarker expression with clinical variables (e.g., age, CA125, RMI). All comparisons were two sided and significance threshold was set to a p-value of less than 0.05. Kaplan Meier analysis was used to determine overall and disease specific survival in relation to TILs expression in the studied cohort. In accordance with the journal's guidelines, we will provide our data for the reproducibility of this study in other centres if such is requested.

## Results

### Patient characteristics

A total of 58 patients fulfilled the initial inclusion criteria, however one was subsequently excluded due to a diagnosis of primary colonic malignancy, giving a total of 57 cases in the cohort. There were 23 women in the borderline cohort and 34 in the invasive cancer cohort. The histological diagnoses and patient characteristics are described in Table I. The median age was 41 (Interquartile range 28.5-46), with over a third of women belonging to the over 45 category. There was no significant difference in age at diagnosis between invasive ovarian cancer and borderline ovarian tumour (BOT) (MWW, P=0.33). The majority of women (89.5%) were of white ethnicity, with representation from black Caribbean, Pakistani and Indian ethnicities included. The most common diagnosis was serous epithelial ovarian cancer (n=21, 36.8%), followed by mucinous BOT and serous BOT. Women largely presented in stage 1 (59.6%) or stage 3 (26.3%) disease.

The median follow up was 56 months (range 1–114 months). A total of 15 women (44%) succumbed to their disease and this was exclusively within the cancer cohort. No deaths were seen in the BOT category. The risk of malignancy index (RMI) was significantly greater among the women diagnosed with cancer (MWW, P=0.04) and the CA125 serum level tended to be higher within the cancer cohort (MWW, P=0.05).

### CD3 expression

**Figure 2.**
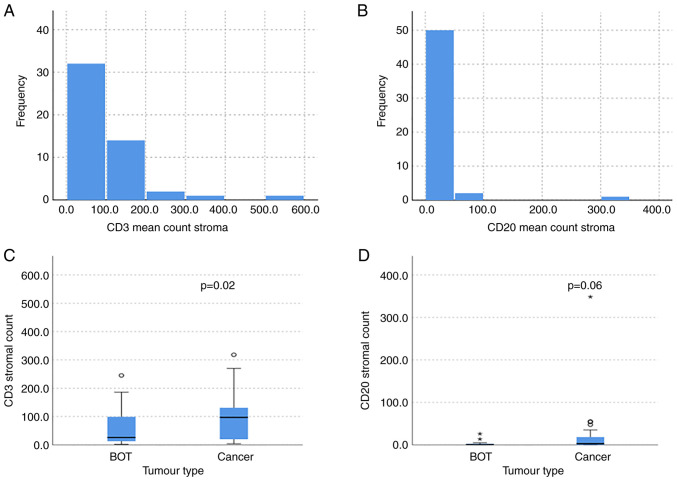
Mean CD3 and CD20 expression within all samples and in ovarian cancer and BOT. (A) Mean infiltration of CD3 cells among all samples (n=57). (B) Mean infiltration of CD20 cells within all samples (n=57). (C) Boxplot graph showing higher median infiltration of CD3-positive TILs in the stroma of cancer compared with BOT (MWW; P=0.02). (D) Boxplot graph showing higher median CD20-positive TILs in the stroma of cancer compared with BOT (MWW; P=0.06). Boxes span the 25th and 75th percentiles. Lines across the boxes represent medians. Circles and asterisks represent outliers and extreme values, respectively. BOT, borderline ovarian tumours; MWW, Mann-Whitney-U Wilcoxon (rank sum) test; TILs, tumour-infiltrating lymphocytes.

Regardless of the underlying histology, we noted that within our cohort there was a greater infiltration of CD3 cells within the stroma compared to CD20 cells. The mean CD3 infiltration was 91.3 (SD 102.4) whilst with CD20 cells the mean was 14.6 (SD 18.7) as shown in Fig. 2A and B. There were significantly more CD3 and CD20 TILs residing in the stroma compared to within the tumour (MWW, P=0.01 and P=0.008). The infiltration of T cells (CD3 positive) was significantly greater within the stroma of cancer cases compared to BOT (MWW, P=0.02) as shown in Fig. 2C. The median CD3 stromal count for both clear cell and endometrioid cancers was 139, compared to 84.5 in serous cancer per 10 high power fields. Within the BOT samples, the median CD3 stromal count was much lower, with 23 and 46.5 for serous and mucinous BOT respectively. When considering the percentage stromal infiltration, the clear cell cancers had the greatest abundance with a median of 25%, compared to 15% in endometrioid, 7% in serous cancer and 5% in mucinous cancer. The percentage stromal infiltration was much greater in cancer compared to BOT, however, as the mucinous BOT had a median percentage of 1.25%, whilst the serous BOT had 0.5%. The mean CD3 stromal and intra-tumoural lymphocyte count did not significantly differ between the different epithelial cancer histological subtypes (KWt, stromal P=0.36 and intra-tumoural P=0.15). The intra-tumoural infiltration of CD3 cells was not significantly greater in cancer compared to BOT (MWW, P=0.25).

### CD20 expression

Overall, the expression of CD20 in various compartments was much less than that of CD3. There was a trend to a greater presence of B cells (CD20 positive) within the stroma of cancerous tumours in comparison to BOT (MWW, P=0.06) as shown in Fig. 2D. The median CD20 stromal infiltration was greatest in endometrioid cancer with a score of 13.8, whilst in serous cancer it was 0 (absent). Within the borderline cancers, mucinous BOT had a median CD20 infiltration of 1 and serous BOT had a median of 0. Similar to our findings with CD3 aggregates, there was no significant difference in the presence of CD20 aggregates within the samples (MWW, P=0.22). The stromal CD20 infiltration was not significantly greater in the different histological subtypes, nor was it significant for intra-tumoural infiltration. The intra-tumoural infiltration of CD20 positive lymphocytes attained significance, with more CD20 TILs present in the cancer samples compared to BOT (MWW, P=0.048).

### Touching lymphocyte

**Figure 3.**
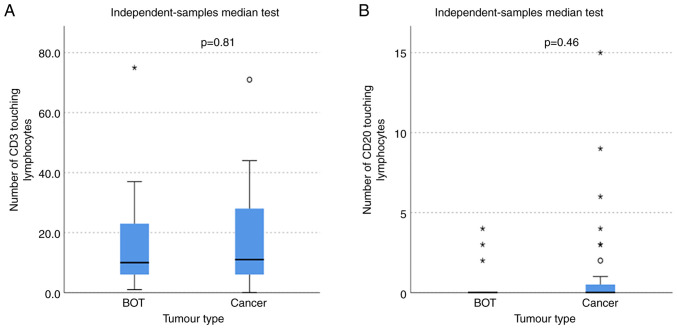
Boxplot graphs of CD3 and CD20 touching lymphocytes. The x-axis represents tumour types and the y-axis represents TIL counts (count of cells). Greater numbers of touching lymphocytes were noted in the cancer samples but the difference was not statistically significant [Mann-Whitney-U Wilcoxon (rank sum) test; CD3, P=0.81; CD20, P=0.46]. (A) Boxplot of CD3 touching lymphocytes in BOT and ovarian cancer. The median number of CD3-positive touching lymphocytes in BOT and cancer was not statistically significantly different. (B) Boxplot of CD20 touching lymphocytes in BOT and ovarian cancer. There was no significant difference in the numbers of CD20-positive touching lymphocytes in BOT and cancer. Boxes span the 25th and 75th percentiles. Lines across the boxes represent medians. Circles represent outliers and asterisks represent extreme values. BOT, borderline ovarian tumours.

The number of T and B lymphocytes within the stroma touching the tumour (touching lymphocytes) was not significantly different between ovarian cancer and BOT (MWW, CD3 P=0.81 and CD20 P=0.46), demonstrated in Fig. 3. In addition, there was no significant difference between the number of touching lymphocytes between the different ovarian cancer subtypes with CD3 cells (KWt, P=0.67) or CD20 cells (KWt, P=0.72).

### TILs aggregates and hotspots

The presence of CD3 aggregates were not significantly different in either the cancer or BOT samples (MWW, P=0.09), nor were there more CD3 hotspots in one cohort over the other (MWW, P=0.81).

### Correlation with patients' outcome

**Figure 4.**
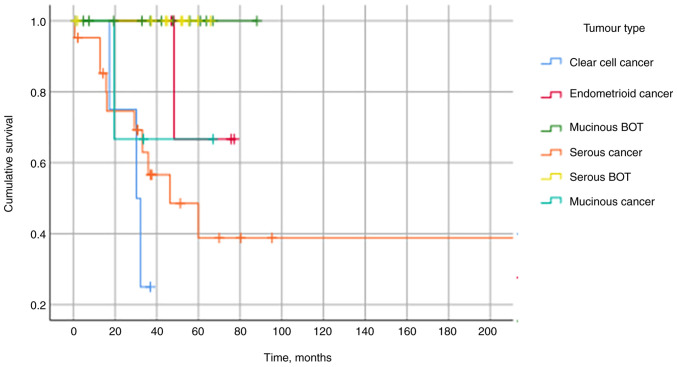
Kaplan-Meier survival curves showing the overall survival of women with BOT and cancer. No deaths occurred in the BOT cohort, indicated by the green and yellow lines. The poorest survival was observed in the clear cell and serous cancer groups. BOT, borderline ovarian tumours.

We evaluated the relationship between the presence of TILs and prognostic indicators. Primarily, all of the deaths occurred within the cancer cohort [n=15/34 (44%) χ^2^, P=0.001] with no mortality within the BOT group. The median survival was 34.8 months in the cancer cohort and mortality did not depend on the underlying histological cancer subtype, with a significance value of P=0.10 (KWt). The infiltration of CD3 or CD20 TILs in the stroma or tumour and the number of aggregates or hotspots were not predictive of mortality. Additionally, the number of touching lymphocytes of CD3 or CD20 cells was not associated with mortality (MWW, P=0.64 and P=0.33 respectively). The Kaplan-Meier curve in Fig. 4 demonstrates no mortality within the BOT cohort and that clear cell carcinoma was associated with the poorest prognosis.

CD3 and CD20 stromal infiltration was significantly correlated with the serum CA125 level (r_s_, P=0.005 and P<0.001 respectively). No statistically significant correlation was found between, intra-tumoural CD3 or CD20 TILs, the presence of aggregates or hotspots and serum CA125.

## Discussion

Our results have primarily demonstrated that there were significantly more CD3 than CD20 TILs in the stroma of our specimens. There were significantly more CD3 TILs within the stroma of ovarian cancers in comparison to BOT and significantly more CD20 within the tumour of cancerous neoplasms in comparison to BOT. Collectively, however, these results failed to impact on prognosis or mortality or response to therapy.

In the context of published literature, this is the first study to analyse the role of TILs in ovarian neoplasms of young women. Our results show a predominant infiltration by CD3 TILs and their greater infiltration within the stroma of ovarian cancer cases compared to those with BOT. This finding is consistent with other literature in ovarian cancer of older women, but also in other solid cancers. Stromal TILs have been found to be prognostic of outcome in triple negative breast cancer and as such, their routine reporting has been proposed (14,15). Within ovarian cancer, this has been previously demonstrated comparing benign and malignant ovarian tumours whereby those which were malignant had a greater stromal infiltration (16). A recent systematic review on TILs in ovarian cancer highlighted a lack of reliable studies investigating stromal CD3 TILs therefore a conclusion was not able to be formed, however intratumoural CD3 TILs were associated with increased progression-free survival and overall survival (17). Our results did not identify a significant relationship between TIL infiltration and prognosis, a finding that has been corroborated in other published work (18). In addition, a comparison between malignant and BOT, to our knowledge, has not been performed previously.

CD20 positive B cells have been shown to be associated with an improved clinical outcome in high grade serous (HGS) cancer (19–21) as well as other cancers including lung, melanoma and breast (22–26). We demonstrated that CD20 infiltration was considerably lower than that of CD3 infiltration in both the stroma and intra-tumoural compartments.

In our cohort, CD20 prevalence was reduced in HGS ovarian cancer in comparison to endometrioid and mucinous cancers. This is in contrast to the findings by Milne *et al* (27) who demonstrated a greater infiltration of CD20 positive cells in HGS cancer. They also found that disease-specific survival was greater in those with greater CD20 infiltration (27). CD20 TILs are thought to have antigen-presenting cell functionality as well as a direct cytolytic response (19) so contribute greatly to the immune microenvironment. The cohort used within that study, however, was considerably older than our cohort. Whether age affects the tumour biology and subsequent immune infiltration is unknown but work is currently underway to investigate this. There are no published studies identifying the differences in stromal infiltration of B cells between the different ovarian cancer subtypes.

Aside from primary tumours, CD20 positive B cells have also been found to be present in omental metastases of HGS ovarian cancer, localising mainly in lymphoid aggregates within the stroma. Their presence was unaffected by the exposure to chemotherapy, however those who had been exposed to neo-adjuvant chemotherapy had a greater proportion of class-switched memory B cells, potentially contributing to an improved response to therapy (28). The presence of stromal CD20 cells has also been associated with the absence of lymph node metastases and therefore may play a role in preventing metastatic spread.

It is known that there is significant heterogeneity of immune cells within the tumour microenvironment between patients and also between different sites in the same patient (29–31). Additionally, the degree of infiltration of CD3+ cells prior to chemotherapy has, however, been shown to vary wildly within HGS cancer patients (21). As such, the interrelationship of both T and B cells should be considered. A meta-analysis demonstrated that the presence of intraepithelial CD3+ and CD8+ lymphocytes improved survival despite significant heterogeneity between the published studies (13).

Despite multiple publications demonstrating correlation of TILs infiltration and prognosis in HGS ovarian cancer and other solid tumours, our results did not corroborate with this in this young age group. This may be due to the population we sampled as we chose a specific population of women presenting with ovarian tumours under the age of 50. As such these women may present atypically and demonstrate a different biology/immune infiltrate when compared to the more common presentation of women in their 5th or 6th decade of life. There has been one study identified which split their cohort into 2; those aged less than 52 and those greater than 52. This did not show any difference in CD3 infiltration between the cohorts, suggesting that age may not be an important factor. They also found that tumour size or its location, presence of ascites, and lymph node metastasis had no significant association with the densities of CD3 cells (32).

In addition to this, our population was rather unique as the vast majority of women presented in stage 1 of their disease. Usually, women tend to present at stage 3 or 4 due to the vague presenting symptoms and late diagnosis. Whether this also has contributed to our findings is unclear; perhaps women presenting in later stage disease have greater stromal and intra-tumoural infiltration compared to those women in stage 1, potentially dictated by their histological sub-type and host genetic and immune factors. Our population was also largely consisting of white women with very few women representing ethnic minority groups. This needs to be considered and further work is required to detect any interracial differences within the population.

We showed that touching lymphocytes were not predictive of histology or prognosis. We assessed this parameter because dense touching lymphocytes in other cancers such as breast cancers have been associated with more aggressive tumours, a shorter recurrence-free interval and ductal carcinoma in situ recurrence. When more traditional methods of scoring were used, such as counting stromal or intra-epithelial TILs, there was considerable variability in association with the outcome. This method of counting was also shown to be superior with regards to reproducibility between assessors (11). Whether touching lymphocytes become more reliable and predictive in the older population with ovarian cancer is unknown.

Our findings suggested that CD3 and CD20 infiltration was greatest in endometrioid cancers but there was no statistically significant difference demonstrated between the different ovarian cancer subtypes or the prognosis. The density of infiltration can vary considerably between patients and even within the same patient, as mentioned above, so it may be necessary to expand our cohort to see if any of results attain significance. It has been found that TIL infiltration was greatest in HGS and endometrioid ovarian cancer in both intra-tumoural and stromal samples, however it was unclear as to the cell types included within the ‘TIL’ definition. Interestingly, our results echo their findings that there was no statistically significant difference in prognosis between those with a greater TIL infiltration and those with few TILs (33). No existing publications were identified that investigated T and B cell infiltration specifically in the various sub-types of ovarian cancer; most focus solely on the most common, HGS cancer. As such this research would be a welcome contribution to existing knowledge.

In accordance with the findings of Toss *et al* (11) in breast carcinoma, we found no significant association between the presence of TILs hotspots or aggregates with prognosis.

We found that the risk of malignancy index (RMI) and CA125 were greater in women with cancer compared to women with a diagnosis of BOT. It has been shown that CA125 has a linear relationship with CD3 infiltration (32), which corresponds to our findings. RMI is used to differentiate cancer from benign lesions (34), however it has also been shown to differentiate benign from borderline tumours (35). There is limited published data concerning the use of RMI to differentiate BOT and ovarian cancer as usually these diagnoses are grouped together and compared to benign tumours.

While this is a retrospective cohort, there are several strengths to this study. The patient cohort is unique (including young women with ovarian cancer and separating borderline and ovarian malignancies; two cohorts that are often grouped together) with a long follow up. There was rigorous histological assessment with slide review, selection of representative tumour blocks, optimal staining following diagnostic standards in a UKAS accredited laboratory and detailed TILs assessment following international guidance and overseen by experienced pathologists. Potential weaknesses with this study lie in its small size of cohort and consequently poor ethnic diversity.

This research has shown an increase in TILs in cancer specimens but that this does not impact on mortality or prognosis, therefore TILs assessment in routine histological assessment is not duly warranted. In addition, the assessment of touching lymphocytes was not predictive of prognosis and as such does not require particular assessment, unlike that advised in the histological assessment of breast carcinoma.

To further this work, it would be prudent to investigate the relationship of both CD3 and CD20 positive cells to other important immunoregulatory cells, such as CD8 T cells and macrophages. It has been extensively demonstrated that CD8 cells are found in tertiary lymphoid structures with CD20 B cells, alongside dendritic cells (20). Tumour-associated macrophages (TAMs) have been found to exhibit an immunomodulatory effect on the tumour microenvironment with M2 subset enabling tumour growth in both the primary and metastatic sites. It has been shown that TAMs have been identified in greater numbers at sites of metastasis in comparison to primary sites, demonstrating their potential role in creating an environment conducive to metastatic deposit and development (31,36). Additionally, further work is required in an older population to determine if our findings remain true in women presenting over the age of 50.

## Figures and Tables

**Table I.** Patient and clinicopathological characteristics of the studied cohort.

Variable	Value
Age at diagnosis, n (%)	
15–19 years	1 (1.8)
20–24 years	6 (10.5)
25–29 years	9 (15.7)
30–34 years	3 (5.3)
35–39 years	5 (8.8)
40–44 years	11 (19.3)
45–49 years	22 (38.6)
Median age (IQR), years	41 (28.5–46.0)
Ethnicity, n (%)	
White British	51 (89.5)
Indian	2 (3.5)
Pakistani	1 (1.8)
Black Caribbean	1 (1.8)
Not stated	2 (3.5)
Histological diagnosis, n (%)	
Clear cell cancer	5 (8.8)
Endometrioid cancer	5 (8.8)
Mucinous cancer	3 (5.3)
Serous cancer	21 (36.8)
Serous BOT	11 (19.3)
Mucinous BOT	12 (21.1)
Stage, n (%)	
1	34 (59.6)
2	3 (5.3)
3	15 (26.3)
4	5 (8.8)
RMI, n (%)	
0–250	22 (38.6)
251–500	7 (12.3)
501–1,000	3 (5.3)
1,001+	14 (24.6)
Not stated	11 (19.3)
Mean RMI ± SD	3,008.6±3,810.7
Chemotherapy, n (%)	
No	25 (43.9)
Yes	32 (56.1)
Recurrence, n (%)	
Yes	15 (26.3)
No	38 (66.6)
Not stated	4 (7.0)
Deceased	
Yes	15 (26.3)^a^
No	42 (73.7)

a From the patients with cancer only, no deaths in the borderline group. BOT, borderline ovarian tumour; RMI, risk of malignancy index.

## Data Availability

The datasets used and/or analyzed during the current study are available from the corresponding author on reasonable request.

## Abbreviations

BOT: borderline ovarian tumour
RMI: Risk of Malignancy Index
TILs: tumour-infiltrating lymphocytes
